# Supplementary material for: Evaluation of a New Monoclonal Chemiluminescent Immunoassay Stool Antigen Test for the Diagnosis of Helicobacter pylori Infection: A Spanish Multicentre Study
Source: J Clin Med. 2022 Aug 29;11(17):5077. doi: 10.3390/jcm11175077 (PMC9457298; doi:10.3390/jcm11175077)
Supplement: Supplementary file 1 [file jcm-11-05077-s001.zip › jcm-1885035-supplementary.pdf]

DiaSorin Inc.
1951 Northwestern Ave – Stillwater, MN 55082 – USA
Tel 1.651.439.9710 – Fax 1.651.351.5669

Changes: §
Deletions: §

LIAISON® Meridian *H. pylori* SA (REF 318200)

1. INTENDED USE

The LIAISON® Meridian *H. pylori* SA assay is a chemiluminescent immunoassay (CLIA) intended for the qualitative determination of *Helicobacter pylori* (*H. pylori*) antigen in human stool. The test is an aid in the diagnosis of patients suspected of H. pylori infection and to measure post therapy response from patients who have discontinued therapy for at least 4 weeks. Assay results should be used in conjunction with other clinical and laboratory data to assist the clinician in making individual patient management decisions.
The test must be performed on the LIAISON® XL Analyzer.

Caution: US Federal Law restricts this device to sale by or on the order of a licensed practitioner.

2. SUMMARY AND EXPLANATION OF THE TEST

Helicobacter pylori is a gram-negative, helix-shaped, bacterium found in the human stomach. It is the causative agent of chronic gastritis or inflammation of the stomach lining, duodenal and stomach ulcers, and is associated with an increased risk of stomach cancer.[1] Although the exact route of transmission is not known; oral-oral and/or fecal-oral routes are generally accepted.[2,3] Once colonization of the stomach is established, *H. pylori* will likely persist indefinitely unless antimicrobial intervention is prescribed.
Unlike a majority of bacterial species, *H. pylori* is capable of colonizing the harsh acidic environment of the stomach. To accomplish this, *H. pylori* uses it's flagella to actively burrow through the mucus reaching the stomach's epithelial cell layer.[4] Additionally *H. pylori* produces urease, which degrades urea into carbon dioxide and ammonia, helping to neutralize the gastric acid present in the stomach.
Several methods are used to diagnose *H. pylori* infection including gastric biopsy, UBT (urea breath test), and stool ELISA. The LIAISON® Meridian *H. pylori* SA assay detects the presence of *H. pylori* antigen in human stool.

3. PRINCIPLE OF THE PROCEDURE

The LIAISON® Meridian *H. pylori* SA assay is a delayed one-step sandwich assay for detection of *H. pylori* stool antigen. The assay uses a monoclonal antibody for detection of *H. pylori* stool antigen. The assay uses 200 µL of sample consisting of a mixture of sample diluent and stool extracted *H. pylori* stool antigen which is incubated with paramagnetic particles coated with a capture antibody for *H. pylori* stool antigen. Following incubation, an isoluminol conjugated antibody for *H. pylori* stool antigen is added to the reaction and incubated. After the second incubation, the unbound material is removed with a wash cycle. The starter reagents are then added and a flash chemiluminescent reaction is initiated. The light signal is measured by a photomultiplier as relative light units (RLU) and is proportional to the concentration of *H. pylori* stool antigen present in the calibrators, controls or samples.

4. MATERIALS PROVIDED

Reagent Integral

Magnetic Particles (2.4 mL)	SORB	Magnetic particles coated with a mouse monoclonal antibody against *H. pylori* stool antigen in phosphate buffer, BSA, surfactant, 0.1% ProClin® 300 and 0.05% gentamicin sulfate
Conjugate (13.0 mL)	CONJ	Mouse Monoclonal antibody conjugated to an isoluminol derivative in phosphate buffer, BSA, surfactant, 0.1% ProClin® 300 and 0.05% gentamicin sulfate
Assay Buffer (13.0 mL)	BUF AS	Mouse IgG in phosphate buffer, BSA, surfactant, 0.1% ProClin® 300 and 0.05% gentamicin sulfate
Number of Tests		100

ProClin is a trademark of the Dow Chemical Company (Dow) or an affiliated company of Dow.

The order of reagents reflects the layout of containers in the reagent integral.

Additional components not on the reagent integral

Calibrator 1<br>2 x 2.0 mL<br>Lyophilized	CAL 1	*H. pylori* stool antigen in phosphate buffer, BSA, surfactant, 0.1% ProClin® 300 and 0.05% gentamicin sulfate. Reconstitute with 2.0 mL distilled or deionized water.
Calibrator 2<br>2 x 2.0 mL<br>Lyophilized	CAL 2	*H. pylori* stool antigen in phosphate buffer, BSA, surfactant, 0.1% ProClin® 300 and 0.05% gentamicin sulfate. Reconstitute with 2.0 mL distilled or deionized water.
Sample Diluent<br>1 x 100 mL	DIL SPE	Phosphate buffer, BSA, surfactant, 0.1% ProClin® 300 and 0.05% gentamicin sulfate<br>After opening, Sample Diluent is stable for 8 weeks when stored at 2-8 °C.
2 x 50 each	PIPETTOR	Liquid Stool Pipettors (REF X0031)
LIAISON® Stool Extraction Device*<br>2 x 50 each part	TUBES<br>FILTERS<br>CAPS	Polypropylene mixing tube, conical tube and blue cap, with high-density polyethylene (HDPE) blue filter unit.

*Device does not contain Bisphenol A (BPA), latex or Di (2-ethylhexyl) phthalate (DEHP).

Standardization: The calibrator concentrations are referenced to an in-house standard preparation.

Materials required but not provided (system related)

**LIAISON® XL Analyzer**
LIAISON® Wash/System Liquid (REF 319100)
LIAISON® XL Waste Bags (REF X0025)
LIAISON® XL Cuvettes (REF X0016)
LIAISON® XL Starter Kit REF 319200)
LIAISON® XL Disposable Tips (REF X0015)

Additional required materials:
LIAISON® Meridian *H. pylori* SA Control Set (REF 318201)

Optional Laboratory Supplies available from DiaSorin
Liquid Stool Pipettors (REF X0031)

5. WARNINGS AND PRECAUTIONS

FOR *IN VITRO* DIAGNOSTIC USE – Not for internal or external use in humans or animals.

General Safety:

- All specimens, biological reagents and materials used in the assay must be considered potentially able to transmit infectious agents. Avoid contact with skin, eyes or mucous membranes. Follow good industrial hygiene practices during testing.
- Do not eat, drink, smoke or apply cosmetics in the assay laboratory.
- Do not pipette solutions by mouth.
- Avoid direct contact with all potentially infectious materials by wearing lab coat, protective eye/face wear and disposable gloves.
- Wash hands thoroughly at the end of each assay.
- Avoid splashing or forming aerosols when handling, diluting or transferring specimens or reagents. Any reagent spill should be decontaminated with 10% bleach solution (containing 0.5% sodium hypochlorite) and disposed of as though potentially infectious.
- Waste materials should be disposed of in accordance with the prevailing regulations and guidelines of the agencies holding jurisdiction over the laboratory, and the regulations of each country.
- Do not use kits or components beyond the expiration date given on the label.

Chemical Hazard and Safety Information: Reagents in this kit are classified in accordance with US OSHA Hazard Communication Standard; individual US State Right-to-Know laws; Canadian Centre for Occupational Health and Safety Controlled Products Regulations; and applicable European Union directives (see Material Safety Data Sheet for additional information).

GHS/CLP:

	ProClin®
CAS No.:	55965-84-9
Reagents:	SORB<br>CONJ<br>BUF AS<br>CAL 1<br>CAL 2<br>DIL SPE
Classification:	Skin sensitization, Category 1
Signal Word:	Warning
Pictogram:	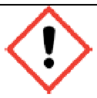<br>GHS07 – Exclamation mark
Hazard Statements:	H317 – May cause an allergic skin reaction.
Precautionary Statements:	P261 – Avoid breathing mist or spray.<br>P272 – Contaminated work clothing should not be allowed out of the workplace.<br>P280 – Wear protective gloves and clothing, and eye protection.

6. REAGENT INTEGRAL PREPARATION

Please note the following important reagent handling precautions:

6.1 Resuspension of magnetic particles

Magnetic particles must be completely resuspended before the integral is placed on the instrument. Follow the steps below to ensure complete suspension:

- Before the seal is removed, rotate the small wheel at the magnetic particle compartment until the colour of the suspension has changed to brown. Gentle and careful side-to-side mixing may assist in the suspension of the magnetic particles (avoid foam formation). Visually check the bottom of the magnetic particle vial to confirm that all settled magnetic particles have resuspended.
- Repeat as necessary until the magnetic particles are completely resuspended.
- After removal of the seal carefully wipe the surface of each septum to remove residual liquid if necessary.

6.2 Foaming of reagents

In order to ensure optimal performance of the integral, foaming of reagents should be avoided. Adhere to the recommendation below to prevent this occurrence:

- Visually inspect the reagents to ensure there is no foaming present before using the integral. If foam is present after re-suspension of the magnetic particles, place the integral on the instrument and allow the foam to dissipate. The integral is ready to use once the foam has dissipated and the integral has remained onboard and mixing.

6.3 Loading of integral into the reagent area

LIAISON® XL Analyzer

LIAISON® XL Analyzer is equipped with a built-in solid-state magnetic device which aids in the dispersal of microparticles prior to placement of a reagent integral into the reagent area of the analyzer. Refer to the analyzer operator's manual for details.

a. Insert the reagent integral into the dedicated slot.
b. Allow the reagent integral to remain in the solid-state magnetic device for at least 30 seconds (up to several minutes). Repeat as necessary.

- Place the integral into the reagent area of the analyzer with the label facing left and let it stand for 15 minutes before using. The analyzer automatically stirs and completely resuspends the magnetic particles.
- Follow the analyzer operator's manual to load the specimens and start the run.

7. STORAGE AND STABILITY OF THE REAGENT INTEGRAL

Upon receipt, the reagent integral must be stored in an upright position to facilitate re-suspension of magnetic particles. When the reagent integral is stored unopened the reagents are stable at 2-8°C up to the expiration date. Do not freeze. The reagent integral should not be used past the expiration date indicated on the kit and reagent integral labels. After removing seals, integrals may be returned to the kit box and stored upright at 2-8°C or stored on board the analyzer for 8 weeks.

8. SPECIMEN COLLECTION AND STORAGE

Collect stool specimens into a clean airtight container with no preservative. Specimens should be stored at 2-8°C upon receipt for up to 72 hours, after this time period the specimen should be stored at -20 °C. Allow specimens to warm to room temperature and mix as thoroughly as possible before use. Test immediately after specimen is warmed to room temperature. Avoid repeated freeze/thaw cycles.

9. SPECIMEN EXTRACT STORAGE

Stool specimen extracts are stable for 8 hours at 18-25°C (room temperature) or 72 hours at 2-8°C (refrigerated) prior to testing. For long term storage, stool specimen extracts may be stored for up to 12 weeks at -20 °C. Frozen stool specimen extracts can be used through 3 freeze-thaw cycles.

Prior to long term storage in refrigerator or freezer, or during transport, extract must be removed from visible debris that may be present at the bottom of the conical tube. Transfer extract to a different sample tube; do not mix visible debris at bottom of conical tube into extract.

10. CALIBRATORS 1 and 2

The LIAISON® Meridian *H. pylori* SA calibrators are supplied lyophilized. Reconstitute each vial with 2.0 mL of distilled or deionized water. Allow the vial(s) to stand for 10 minutes at room temperature, mix gently by inversion until completely dissolved. Ensure any lyophilized material adherent to vial stopper is also dissolved. Transfer a minimum of 750 µL (triplicate calibration) to a glass or plastic sample tube. Affix the appropriate bar code label to the tube. Place onto the analyzer. In case external calibrator barcodes fail to be read, data present on the external calibrator labels (under the barcode) may be manually entered on the analyzer. Calibrate the assay as described in the analyzer operator's manual.

LIAISON® Meridian *H. pylori* SA calibrators should be aliquoted after reconstitution if not assayed immediately . LIAISON® Meridian *H. pylori* SA calibrators have been shown to be stable for 8 hours when stored at room temperature and 28 days when stored at 2-8°C. Remaining reconstituted calibrators should be aliquoted to a minimum of 750 µL and can be stored frozen at -20°C for 16 weeks. Frozen calibrators can be used through 3 freeze-thaw cycles. Mix gently by inversion after freeze-thaw cycle prior to use.

Calibrator and reagent integral lot number are lot specific. Do not use calibrators matched with a different reagent lot in the same assay.

11. CALIBRATION

Individual LIAISON® Meridian *H. pylori* SA reagent integrals contain specific information for calibration of the particular reagent integral lot. Test of assay specific calibrators allows the detected relative light units (RLU) values to adjust the assigned master curve. Each calibration solution allows **2** calibrations to be performed.
Recalibration in triplicate is mandatory whenever at least 1 of the following conditions occurs:

- With each new lot of reagents (reagent integral or starter reagents).
- The previous calibration was performed more than 4 weeks prior.
- Quality control results are out of the acceptable range.
- The analyzer has been serviced.

Refer to the analyzer operator's manual for calibration instructions.

Measuring range: The LIAISON® Meridian *H. pylori* SA assay measures between 0.01 and 50 (Index value).
The lowest reportable value is 0.01 Index. Values below 0.01 Index should be reported as < 0.01 Index.
Values above 50 Index should be reported as > 50 Index.

12. SPECIMEN PREPARATION

Using LIAISON® Stool Extraction Device:
Sample and sample diluent volumes should be determined from Table 1 and the diagrams below.

1. Add LIAISON® Sample Diluent C into LIAISON® Stool Extraction Device mixing tube according to Table 1.
2. Stool preparation: Mix stool as thoroughly as possible prior to withdrawing sample.
   a. ***Liquid or Semi-Solid Stools:*** Using disposable liquid stool pipettor, measure and transfer stool volume (see Table 1) into the LIAISON® Stool Extraction Device mixing tube containing the sample diluent. Rinse the pipettor several times with stool suspension mixture if necessary to ensure as much sample as possible is removed from the Liquid Stool Pipettor.
   NOTE: If 750 µL of liquid or semi-solid stool is not available, a 1:1 ratio of stool sample to sample diluent can be used. (Example: 400 µL liquid or semi-sold stool sample to 400 µL sample diluent). Final supernatant volume must be 500 µL in order to perform the single test assay.
   b. ***Solid Stools:*** Using the LIAISON® Stool Extraction Device scoop on the blue conical filter unit, measure and transfer ½ scoop of stool sample (see Table 1 and diagrams below) into the mixing tube containing LIAISON® Sample Diluent C.

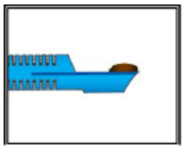
Correct ½ Scoop Stool

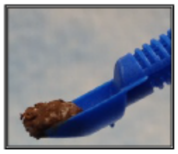
Correct ½ Scoop Stool

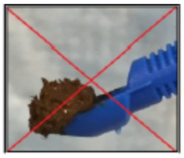
Incorrect ½ Scoop

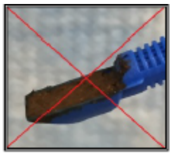
Incorrect ½ Scoop

The LIAISON® Stool Extraction Device scoop should be examined after the vortexing step below (Step 4), to ensure that the solid pellet has been rinsed off the scoop into the sample diluent, otherwise tap the bottom of the device on the bench to aid in release of the stool. This step may be repeated as necessary. Very hard stools may need to be delivered by an alternate device to the sample diluent in the mixing tube.

3. Firmly screw the conical blue filter unit onto the mixing tube.
4. Vortex vigorously for 20 seconds to mix stool thoroughly.
5. Centrifuge tube in a swing bucket centrifuge at a speed of ≥ 2000 x g* for 5 minutes at ambient temperature **with conical end of tube pointing up.**
6. After centrifugation, remove tube and invert LIAISON® Stool Extraction Device so **conical tube is pointing down**. Centrifuge tube at a speed of 200 x g* for 1 minute. **Device must now remain in upright position**.
7. Unscrew the mixing tube and blue filter device and discard into appropriate biohazard waste receptacle.
8. Examine liquid supernatant in conical tube, stool supernatant may be cloudy but no visible debris or bubbles should be present.
9. Place conical tube into LIAISON® XL Analyzer sample rack type "H" with adapter. Load the rack onto the analyzer for testing.
10. Clean work area with 10% bleach solution (0.5% sodium hypochlorite).

Table S1

Sample Type	Sample Diluent Volume	Sample Diameter/ Sample Volume
Solid	1.0 mL	½ scoop (5 mm)
Liquid	750 µL	750 µL

Diagrams illustrating stool preparation procedure using the LIAISON® Stool Extraction Device are provided at the end of the instructions for use.

13. ASSAY PROCEDURE

To ensure proper test performance, strictly adhere to the operating instructions of the analyzer.

LIAISON® XL Analyzer: Each test parameter is identified via information encoded in the Reagent Integral Radio Frequency Identification transponder (RFID Tag). In the event that the RFID Tag cannot be read by the analyzer, the integral cannot be used. Do not discard the reagent integral: contact your local DiaSorin technical support for instruction.

For details, refer to the analyzer operator's manual.

The analyzer operations are as follows:
1. Dispense sample, calibrator or control into reaction module.
2. Dispense magnetic particle and assay buffer into reaction module.
3. Incubate
4. Dispense conjugate
5. Incubate
6. Wash with wash/system liquid
7. Add the starter reagents and measure the light emitted

*g = (1118×10^{-8})(radius in cm)$(rpm)^2$

14. QUALITY CONTROL

Quality control is required to be performed once per day of use, or according to the guidelines or requirements of local regulations or accredited organizations. It is recommended that the user refer to CLSI C24-A3 and 42 CFR 493.1256 (c) for guidance on appropriate quality control practices.

LIAISON® Meridian *H. pylori* SA controls are intended to monitor for substantial reagent failure. LIAISON® controls should be run in singlicate to monitor the assay performance. If control values lie within the expected ranges provided on the certificate of analysis, the test is valid. If control values lie outside the expected ranges, the test is invalid and patient results cannot be reported. Assay calibration should be performed if a control failure is observed and controls and patient specimens must be repeated.

The performance of other controls should be evaluated for compatibility with this assay before they are used. Appropriate value ranges should be established for all quality control materials used.

The range of concentrations of each control is reported on the certificate of analysis and indicates the limits established by DiaSorin for control values that can be obtained in reliable assay runs.

15. INTERPRETATION OF RESULTS

The analyzer automatically calculates *H. pylori* stool antigen levels expressed as Index values and grades the results. For details, refer to the analyzer operator's manual.

The cut-off for the LIAISON® Meridian *H. pylori* SA assay was determined based on the results of testing samples that represented patient populations negative and positive for *H. pylori* stool antigen.

The cut-off value discriminating between the presence and the absence of *H. pylori* stool antigen was determined to have an Index value of 1.0.

Warning – If the sample result displays "invalid RLU" and an exclamation mark (!) flag, the result obtained lies below the assay signal range. The sample must be retested. If the sample upon retest still displays "invalid RLU", call DiaSorin Technical Support.

Patient results should be interpreted as follows:

Index	Results	Interpretation
<0.90	Negative	Indicates the absence of *H. pylori* stool antigen, (or the level of antigen is below that which can be detected by the assay)
≥0.90 and <1.10	Equivocal	Equivocal samples should be retested using a new extraction from the original sample in order to confirm the initial result.<br>Samples that are positive (≥ 1.10) by the second test should be considered positive.<br>Samples that are negative (< 0.90) by the second test should be considered negative.<br>**For samples that are equivocal on retesting; a new specimen should be collected and tested.**
≥1.10	Positive	Indicates the presence of detectable *H. pylori* stool antigen.

Note: The magnitude of the reported Index value is not indicative of the amount of *H. pylori* stool antigen present in the patient sample.

16. LIMITATIONS OF THE PROCEDURE

1. Assay results should be utilized in conjunction with other clinical and laboratory data to assist the clinician in making individual patient management decisions.
2. A skillful technique and strict adherence to the instructions are necessary to obtain reliable results.
3. Antimicrobials, proton pump inhibitors and bismuth preparations are known to suppress *H. pylori* and if ingested may give a false negative result, these medications are known to inhibit *H. pylori*. In these cases, a new fecal sample should be collected and tested 14 days after treatment has stopped. Positive results from patients that have used antibiotics, PPIs, or bismuth compounds in the 14 days prior to fecal sample collection are still considered accurate.
4. A negative test result does not preclude the possibility of the presence of *H. pylori* antigen in the specimen which may occur if the level of antigen is below the detection limit of the test.
5. The LIAISON® Meridian *H. pylori* SA assay has not been evaluated in a pediatric population.
6. Fecal specimens preserved in 10% formalin, Merthiolate formalin, sodium acetate formalin, or polyvinyl alcohol, or specimens that are in transport media such as Cary Blair or C&S cannot be used.
7. Transferring too little sample, or failure to mix and completely suspend the sample mixture, may result in a false-negative test result.

17. EXPECTED VALUES

A study was performed with the LIAISON® Meridian *H. pylori* SA assay testing 277 prospectively collected stool samples from adult subjects who underwent EGD with signs and symptoms of a *Helicobacter* pylori infection. Collection was across gender, known ages ranged from 22 to 87 years of age, and from multiple US and OUS geographical locations. Results showed that a total of 67 subjects were positive with the assay.

The observed prevalence of the LIAISON® Meridian *H. pylori* SA assay is 24.2%. Prevalence may vary depending upon geographical location, age, gender, type of test employed, specimen collection and handling procedures as well as clinical history of the patient.

18. SPECIFIC PERFORMANCE CHARACTERISTICS

18.1 CLINICAL SENSITIVITY and SPECIFICITY

18.1.1 INITIAL DIAGNOSIS

A prospective study consisting of 277 subjects undergoing evaluation to determine *H. pylori* infection status prior to any therapeutic intervention was performed to compare the performance of the LIAISON® Meridian *H. pylori* SA assay to the established composite reference method which is endoscopic biopsy followed by histopathological evaluation, culture, and urease detection test.

Results from stool samples tested by the LIAISON® Meridian *H. pylori* SA assay compared to at least two of the three tests comprising the composite reference method used to determine patient infection status are summarized in the table below.

	Comparator Composite Reference Method		
LIAISON® Meridian *H. pylori* SA	Infected	Not Infected	Total
Positive	64	3	67
Equivocal	0	0	0
Negative	3	207	210
Total	67	210	277

			95% Confidence Interval
Clinical Specificity	207/210	98.6%	95.9 – 99.7%
Clinical Sensitivity	64/67	95.5%	87.5 – 99.1%

18.1.2 POST ERADICATION THERAPY

A prospective study consisting of 8 subjects undergoing evaluation of post therapy response was performed to compare the performance of the LIAISON® Meridian *H. pylori* SA assay to the established composite reference method which is endoscopic biopsy followed by histopathological evaluation, culture, and urease detection test.

Results from stool samples tested by the LIAISON® Meridian *H. pylori* SA assay compared to at least two of the three tests comprising the composite reference method to determine patient infection status are summarized in the table below.

	Comparator Composite Reference Method		
LIAISON® Meridian *H. pylori* SA	Infected	Not Infected	Total
Positive	8	0	0
Equivocal	0	0	0
Negative	0	0	0
Total	8	0	8

			95% Confidence Interval
Clinical Sensitivity	8/8	100%	63.1 – 100%

18.2 PRECISION

A within-laboratory precision study was performed consulting CLSI document EP5-A3 in the preparation of the testing protocol. Six contrived antigen samples containing high negative, low positive and moderate positive concentrations of *H. pylori* stool antigen and kit controls (negative and positive) were assayed in duplicate, in 2 runs per day over 12 operating days with multiple technicians. The following within-laboratory precision results were obtained from samples tested internally at DiaSorin Inc. in 1 kit lot using 1 LIAISON® XL Analyzer.

Sample ID N=48	Mean Index	Within Run		Within Day		Between Day		Total	
		SD	%CV	SD	%CV	SD	%CV	SD	%CV
Neg Ctrl	0.06	0.00	7.8%	0.00	0.0%	0.00	4.6%	0.00	7.8%
Neg Ctrl	0.06	0.00	7.6%	0.00	0.0%	0.00	5.6%	0.01	8.8%
Pos Ctrl	2.72	0.05	1.9%	0.04	1.5%	0.03	1.2%	0.07	2.6%
Pos Ctrl	2.70	0.06	2.4%	0.03	1.2%	0.01	0.2%	0.07	2.7%
Sample #1	0.80	0.02	2.6%	0.02	2.3%	0.03	3.1%	0.04	4.7%
Sample #2	0.84	0.02	2.9%	0.01	1.0%	0.03	3.8%	0.04	4.9%
Sample #3	1.84	0.06	3.1%	0.02	1.2%	0.04	2.4%	0.08	4.1%
Sample #4	1.99	0.04	2.1%	0.07	3.5%	0.02	1.0%	0.08	4.2%
Sample #5	3.03	0.08	2.7%	0.00	0.0%	0.07	2.2%	0.10	3.3%
Sample #6	3.00	0.08	2.6%	0.06	2.1%	0.06	2.0%	0.12	3.9%

A reproducibility/precision study was performed at 2 external sites and internally at DiaSorin Inc. consulting CLSI document EP15-A3 in the preparation of the testing protocol. 6 contrived antigen samples containing high negative, low positive and moderate positive concentrations of *H. pylori* stool antigen and kit controls (negative and positive) were assayed in replicates of 3, in 2 runs per day over 5 operating days with 2 technicians at each site performing the test every day. The following reproducibility/precision results were obtained from samples tested at the 3 sites in 1 kit lot.

Sample ID	Mean Index Value	Within Run		Run to Run Within Day		Day to Day Within Site		Site to Site		Total	
		SD	%CV	SD	%CV	SD	%CV	SD	%CV	SD	%CV
Neg Ctrl	0.075	0.004	5.1%	0.002	2.4%	0.002	2.1%	0.009	12.5%	0.010	13.9%
Neg Ctrl	0.074	0.003	4.0%	0.003	4.0%	0.001	1.4%	0.007	10.0	0.009	11.5%
Pos Ctrl	4.799	0.076	1.6%	0.050	1.0%	0.063	1.3%	0.105	2.2%	0.153	3.1%
Pos Ctrl	4.779	0.070	1.5%	0.050	1.0%	0.068	1.4%	0.113	2.4%	0.157	3.3%
Sample #1	2.118	0.034	1.6%	0.038	1.8%	0.108	5.1%	0.119	5.6%	0.168	8.0%
Sample #2	2.371	0.049	2.1%	0.046	1.9%	0.156	6.6%	0.226	9.5%	0.283	11.9%
Sample #3	0.688	0.024	3.5%	0.021	3.0%	0.037	5.4%	0.065	9.4%	0.081	11.8%
Sample #4	0.695	0.023	3.3%	0.026	3.8%	0.019	2.7%	0.065	9.4%	0.077	11.0%
Sample #5	1.211	0.031	2.5%	0.037	3.1%	0.029	2.4%	0.093	7.7%	0.109	9.0%
Sample #6	1.195	0.021	1.7%	0.030	2.5%	0.056	4.7%	0.120	10.1%	0.138	11.5%

N = 90

18.3 LIMIT OF DETECTION (LoD)

The limit of detection for *H. pylori* stool antigen is 4.0 ng/mL in the LIAISON® Meridian *H. pylori* SA assay.

18.4 INTERFERING SUBSTANCES

Controlled studies of potentially interfering substances from commonly used medications relevant to digestive complications and endogenous interferents spiked into low positive and high negative *H. pylori* antigen stool specimens showed no interference at the concentration for each substance listed below in the LIAISON® Meridian *H. pylori* SA assay. The testing was based on CLSI-EP7-A2.

Substance	Concentration Tested
Barium Sulfate	5.0 mg/mL
Stearic Acid	2.65 mg/mL
Palmitic Acid	1.3 mg/mL
Hemoglobin	3.2 mg/mL
Imodium® AD	6.67×10^{-3} mg/mL
Kaopectate	0.87 mg/mL
Metronidazole	12.5 mg/mL
Mucin	3.33 mg/mL
Mylanta (Maalox®)	4.2 mg/mL
Pepto Bismol®	0.87 mg/mL
MiraLAX® (PEG 3350)	79.05 mg/mL
Prilosec	0.5 mg/mL
Gas X® / Simethicone	0.625 mg/mL
Tagamet	0.5 mg/mL
Tums®	0.5 mg/mL
Vancomycin Hydrochloride	2.5 mg/mL
White Blood cells	5%
Whole Blood	25%

18.5 CROSS REACTIVITY

Assay specificity of the LIAISON® Meridian *H. pylori* SA assay was determined by testing the following microorganisms. Low positive and high negative *H. pylori* antigen stool extracts were spiked with each microorganism and tested by the LIAISON® Meridian *H. pylori* SA assay.
The following organisms did not show interference when spiked into the low positive and high negative stool extracts.

Microorganism (in alphabetical order)	Final conc. of variant in sample		Microorganism (in alphabetical order)	Final conc. of variant in sample
Aeromonas hydrophila	1.2×10^{8} CFU/mL		*Plesiomonas shigelloides*	1.2×10^{8} CFU/mL
Bacillus subtilis	1.2×10^{8} CFU/mL		*Proteus vulgaris*	1.2×10^{8} CFU/mL
Borrellia burgdorferi	1.2×10^{8} CFU/mL		*Pseudomonas aeruginosa*	1.2×10^{8} CFU/mL
Campylobacter coli	1.2×10^{8} CFU/mL		*Pseudomonus fluorescens*	1.2×10^{8} CFU/mL
Campylobacter fetus	1.2×10^{8} CFU/mL		*Salmonella Group B*	1.2×10^{8} CFU/mL
Campylobacter jejuni	1.2×10^{8} CFU/mL		*Salmonella Group C*	1.2×10^{8} CFU/mL
Campylobacter upsaliensis	1.2×10^{8} CFU/mL		*Salmonella Group D*	1.2×10^{8} CFU/mL
Campylobacter hyointestinalis	1.2×10^{8} CFU/mL		*Salmonella Group E*	1.2×10^{8} CFU/mL
Candida albicans	1.2×10^{8} CFU/mL		*Serratia liquefaciens*	1.2×10^{8} CFU/mL
Citrobacter freundii	1.2×10^{8} CFU/mL		*Shigella boydii*	1.2×10^{8} CFU/mL
Clostridium difficile	1.2×10^{8} CFU/mL		*Shigella flexneri*	1.2×10^{8} CFU/mL
Clostridium perfringens	1.2×10^{8} CFU/mL		*Shigella sonnei*	1.2×10^{8} CFU/mL
Clostridium sordellii	1.2×10^{8} CFU/mL		*Staphylococcus aureus*	1.2×10^{8} CFU/mL
Enterobacter cloacae	1.2×10^{8} CFU/mL		*Staphylococcus epidermidis*	1.2×10^{8} CFU/mL
Enterococcus faecalis	1.2×10^{8} CFU/mL		*Vibrio parahaemolyticus*	1.2×10^{8} CFU/mL
Escherichia coli	1.2×10^{8} CFU/mL		*Yersinia enterocolitica*	1.2×10^{8} CFU/mL
Escherichia fergusonii	1.2×10^{8} CFU/mL		Adenovirus Type 2	$1 \times 10^{5.06}$ $TCID_{50}$/mL
Escherichia hermannii	1.2×10^{8} CFU/mL		Adenovirus Type 40	$1 \times 10^{5.06}$ $TCID_{50}$/mL
Haemophilus influenzae	1.2×10^{8} CFU/mL		Adenovirus Type 41	$1 \times 10^{5.06}$ $TCID_{50}$/mL
Klebsiella pneumonia	1.2×10^{8} CFU/mL		Coxsackievirus B1	$1 \times 10^{5.06}$ $TCID_{50}$/mL
Lactobacillus lactis	1.2×10^{8} CFU/mL		Coxsackievirus B6	$1 \times 10^{5.06}$ $TCID_{50}$/mL
Listeria monocytogenes	1.2×10^{8} CFU/mL		Echovirus	$1 \times 10^{5.06}$ $TCID_{50}$/mL
Peptostreptococcus anaerobius	1.2×10^{8} CFU/mL		Rotavirus	$1 \times 10^{5.06}$ $TCID_{50}$/mL

18.6 HIGH DOSE HOOK EFFECT

No high dose hook effect was observed for *H. pylori stool* antigen concentrations measured at >50 Index values.

19. REFERENCES

1. Kusters JG, van Vliet AH, Kuipers EJ (2006). Pathogenesis of *Helicobacter pylori* Infection. Clin Microbiol Rev 19(3):449-90.

2. Brown LM (2000). *Helicobacter pylori*: epidemiology and routes of transmission. Epidemiol Rev 22(2):283-97.

3. Mégraud F (1995). Transmission of *Helicobacter pylori*: faecal-oral versus oral-oral route. Aliment Pharmacol Ther 9 Suppl 2:85-91.

4. Amieva MR, El-Omar EM (2008). Host-bacterial interactions in *Helicobacter pylori* infection. Gastroenterology 134(1):306-23.

5. Clinical and Laboratory Standards Institute (CLSI) C24-A3, Vol.26, No.25, Statistical Quality Control for Quantitative Measurements: Principles and Definitions; Approved Guideline - Third Edition.

6. Clinical and Laboratory Standards Institute (CLSI) EP5-A3, Vol.34, No.13, Evaluation of Precision Performance of Quantitative Measurement Methods; Approved Guideline – Third Edition.

7. Clinical and Laboratory Standards Institute (CLSI) EP15-A3, Vol.28, No. 3, User Verification of Precision and Estimation of Bias; Approved Guideline - Third Edition.

8. Clinical and Laboratory Standards Institute (CLSI) EP7-A2, Vol.25, No.27, Interference Testing in Clinical Chemistry; Approved Guideline - Second Edition.

For Customer Service in the US call toll free: 1-800-328-1482

DiaSorin Inc.
1951 Northwestern Avenue
Stillwater, MN 55082-0285

1. LIAISON® Meridian *H. pylori* SA Stool Preparation Using DiaSorin LIAISON® Stool Extraction Device

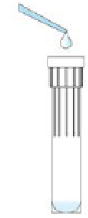

Add LIAISON® Sample Diluent C into mixing tube
A) Liquid or Semi-Solid : Add 750 µL

B) Solid: Add 1.0 mL

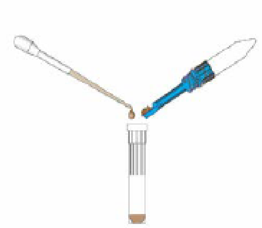

Add Stool:
A) Liquid or Semi-Solid: Add 750 µL using disposable liquid stool pipettor

B) Solid: Add 5 mm (1/2 scoop) using the blue scoop on the conical tube with blue filter unit.

2. Device Assembly

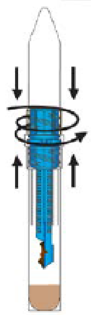

FIRMLY screw the conical blue filter unit onto the mixing tube The outer edge of each should touch.

NOTE: No gap should be visible when device is properly assembled.

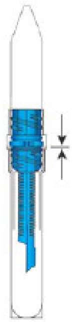

Correct:
No Gap

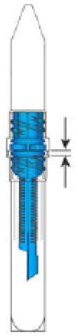

Incorrect:
Gap is visible

3. Mix

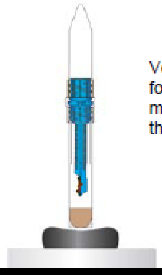

Vortex vigorously for 20 seconds to mix stool thoroughly.

4. Centrifugation

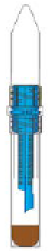

Centrifuge with conical tube pointing up @ ≥ 2000 x g for 5 minutes using a swing bucket centrifuge.

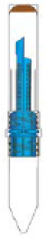

Invert device so conical tube is pointing down. Centrifuge 200 x g for 1 minute.

NOTE: Device must now remain in an upright position.

5. Examination and Testing

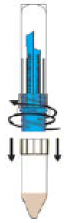

Unscrew conical tube from device.

Discard mixing tube / blue filter unit into appropriate biohazard waste receptacle according to local regulations.

Examine supernatant. Supernatant may be cloudy but no visible debris or bubbles should be present.

Place conical tube on appropriate DiaSorin analyzer for testing or see Section 9 in Instructions for Use for recommended storage.

Clean work area with 10% bleach solution (0.5% sodium hypochlorite).

Biohazard
Single Use Device

ANNEX III

Outcomes Table

Urea Breath Test[1]

$\Delta \leq 4$	$4 < \Delta < 5$	$5 \leq \Delta$
NEG	UNCLEAR<br>Repeat[2]<br>(4 weeks later)	POS

CLIA

<0.90 Index	0,9-1,1	≥1.10 Index
NEG	Equivocal<br>Retested using a new extraction from the original sample	POS

[1] Urea Breath test is the Gold Standard.
[2] A new stool sample must be obtained for the same date.
